# *Pseudomonas aeruginosa nfuA*: Gene regulation and its physiological roles in sustaining growth under stress and anaerobic conditions and maintaining bacterial virulence

**DOI:** 10.1371/journal.pone.0202151

**Published:** 2018-08-09

**Authors:** Adisak Romsang, Jintana Duang-nkern, Kritsakorn Saninjuk, Paiboon Vattanaviboon, Skorn Mongkolsuk

**Affiliations:** 1 Department of Biotechnology, Faculty of Science, Mahidol University, Bangkok, Thailand; 2 Center for Emerging Bacterial Infections, Faculty of Science, Mahidol University, Bangkok, Thailand; 3 Laboratory of Biotechnology, Chulabhorn Research Institute, Bangkok, Thailand; 4 Program in Applied Biological Science: Environmental Health, Chulabhorn Graduate Institute, Chulabhorn Royal Academy, Bangkok, Thailand; 5 Center of Excellence on Environmental Health and Toxicology, Bangkok, Thailand; Centre National de la Recherche Scientifique, Aix-Marseille Université, FRANCE

## Abstract

The role of the *nfuA* gene encoding an iron-sulfur ([Fe-S]) cluster-delivery protein in the pathogenic bacterium *Pseudomonas aeruginosa* was investigated. The analysis of *nfuA* expression under various stress conditions showed that superoxide generators, a thiol-depleting agent and CuCl_2_ highly induced *nfuA* expression. The expression of *nfuA* was regulated by a global [2Fe-2S] cluster containing the transcription regulator IscR. Increased expression of *nfuA* in the Δ*iscR* mutant under uninduced conditions suggests that IscR acts as a transcriptional repressor. In vitro experiments revealed that IscR directly bound to a sequence homologous to the *Escherichia coli* Type-I IscR-binding motifs on a putative *nfuA* promoter that overlapped the -35 element. Binding of IscR prevented RNA polymerase from binding to the *nfuA* promoter, leading to repression of the *nfuA* transcription. Physiologically, deletion of *nfuA* reduced the bacterial ability to cope with oxidative stress, iron deprivation conditions and attenuated virulence in the *Caenorhabditis elegans* infection model. Site-directed mutagenesis analysis revealed that the conserved CXXC motif of the Nfu-type scaffold protein domain at the N-terminus was required for the NfuA functions in conferring the stress resistance phenotype. Furthermore, anaerobic growth of the Δ*nfuA* mutant in the presence of nitrate was drastically retarded. This phenotype was associated with a reduction in the [Fe-S] cluster containing nitrate reductase enzyme activity. However, NfuA was not required for the maturation of [Fe-S]-containing proteins such as aconitase, succinate dehydrogenase, SoxR and IscR. Taken together, our results indicate that NfuA functions in [Fe-S] cluster delivery to selected target proteins that link to many physiological processes such as anaerobic growth, bacterial virulence and stress responses in *P*. *aeruginosa*.

## Introduction

*Pseudomonas aeruginosa* is one of the most common gram-negative bacteria causing nosocomial infections with high mortality worldwide. During the host-microbe interaction, *P*. *aeruginosa* encounters oxidative stress generated either from phagocytic cells of the host innate immune response or from the killing mechanism of some bactericidal antibiotics [1]. Thus, the bacterial ability to endure oxidative stress is crucial for the survival of this pathogen during infection.

[Fe-S] clusters are ubiquitous prosthetic groups for numerous proteins that participate in various fundamental cellular activities, including gene regulation, iron/sulfur storage, aerobic and anaerobic respiration, and biosynthetic pathways [2]. To date, three types of [Fe-S] cluster biosynthetic machineries have been discovered in prokaryotes, i.e., ISC (iron-sulfur cluster), SUF (sulfur formation), and NIF (nitrogen fixation) (for a review see [2, 3]). The ISC and SUF systems contribute to the biogenesis of [Fe-S] clusters that are supplied for the maturation of most proteins in the cell; however, the NIF system is required for maturation of nitrogenase in nitrogen-fixing bacteria [4, 5]. *P*. *aeruginosa* possesses ISC machinery encoded by the *iscRSUA*-*hscBA*-*fdx2-iscX* gene cluster, which is responsible for [Fe-S] cluster biosynthesis [6]. IscR acts as a transcriptional repressor of the *isc* operon. Holo-IscR, a form containing a [2Fe-2S] cluster, directly binds to Type-I IscR-binding motifs located in the vicinity of the promoter and modulates *isc* gene expression [6].

NfuA was first reported in *Escherichia coli* as a [Fe-S] scaffolding protein required for maturation of a [Fe-S] cluster containing proteins under oxidative stress and iron starvation conditions [7] as well as in *Azotobacter vinelandii* as an intermediate [Fe-S] cluster carrier protein involved in [Fe-S] protein maturation [8]. A recent report in *E*. *coli* showed that NfuA is a non-SUF and non-ISC [4Fe-4S] carrier protein that is involved in maturation of [Fe-S] enzymes such as aconitase B and NADH dehydrogenase [9]. NfuA has two functionally related domains. The N-terminal domain is homologous to the ATC-type [Fe-S] carrier domain, which often has a variant of the CXXC domain that lacks the three C residues involved in the ligand for the cluster. The C-terminal domain is homologous to the Nfu domain of NifU, a U-type scaffold protein dehydrogenase involved in the assembly and transfer of [4Fe-4S] to target proteins [2, 9]. The physiological function of NfuA in *P*. *aeruginosa* is as yet unclear. Mutation of *nfuA* renders *P*. *aeruginosa* more susceptible to fluoroquinolone antibiotics than the wild type, under aerobic growth [10]. Here, we further investigated the gene regulation and the function of *P*. *aeruginosa nfuA*. The expression profile of *nfuA*, which could be induced in response to exposure to various oxidants together with the oxidant-vulnerable phenotypes of the *nfuA* mutant, suggests the contribution of NfuA to the protection of *P*. *aeruginosa* against oxidative stress. The induction of *nfuA* expression against the stress response was dependent on transcriptional control through [2Fe-2S] cluster-ligated IscR. Moreover, the *nfuA* mutant exhibited dramatically retarded growth under anaerobic conditions using nitrate as an electron due to the decrease in nitrate reductase activity level and showed attenuated virulence in a *C*. *elegans* host model system.

## Results and discussion

### The expression patterns of *P*. *aeruginosa nfuA* under stress conditions

**Fig 1:**
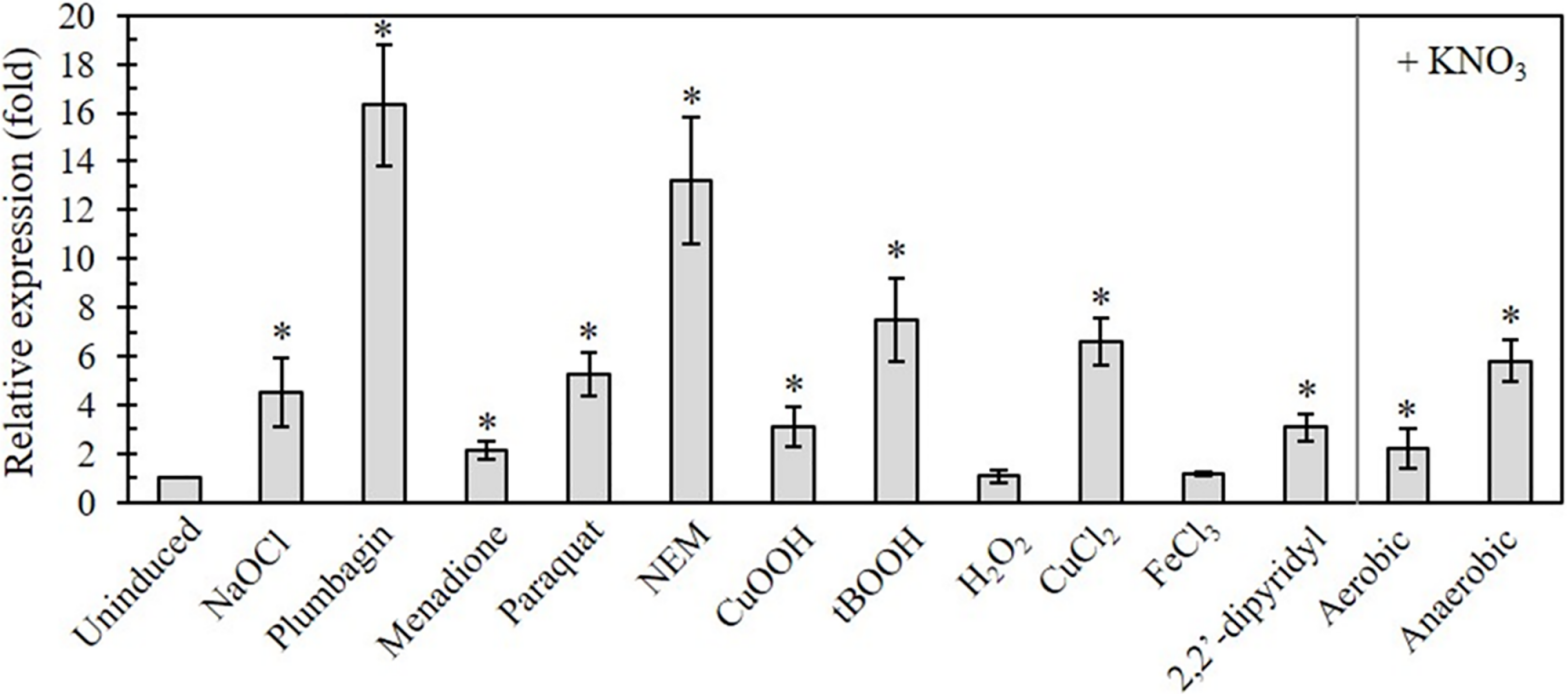
*nfuA* expression in response to stress conditions. Analysis of the *nfuA* expression during normal growth and in response to oxidants. PAO1 and various mutant cultures were treated with 0.02% NaOCl, 0.5 mM Plumbagin, 0.5 mM Menadione, 0.5 mM Paraquat, 0.1 mM NEM, 1 mM CuOOH, 1 mM tBOOH, 1 mM H_2_O_2_, 0.5 mM CuCl_2_, 0.5 mM FeCl_3_, and 1 mM 2,2’-dipyridyl for 15 min under aerobic conditions. For anaerobic growth conditions, LB supplemented with nitrate (+ KNO_3_) was used. RNA extractions and qRT-PCR were performed as described in the Materials and Methods. The data are presented as the means ± SD from three independent experiments. The relative expression was analyzed using the *16S rRNA* gene as the normalizing gene and is presented as the fold expression relative to the level of the uninduced condition.

The expression of genes that contribute to protection against stress conditions is frequently induced in response to such stresses. We initially examined the effects of oxidative stress-generating agents including plumbagin (PB), menadione (MD) and paraquat (PQ) for the intracellular generation of superoxide anions, a thiol-depleting agent, N-ethylmaleimide (NEM), peroxides (cumene hydroperoxide [CuOOH], *tert*-butyl hydroperoxides [tBOOH], hydrogen peroxide [H_2_O_2_]) and sodium hypochlorite (NaOCl) on *nfuA* expression. Treatment of PAO1 with subgrowth inhibitory concentrations of oxidants induced the expression of *nfuA* (PB [16.3 ± 2.5-fold], MD [2.1 ± 0.4-fold], PQ [5.3 ± 0.9-fold], CuOOH [3.1 ± 0.8-fold], tBOOH [7.5 ± 1.7-fold] and NaOCl [5.1± 0.7-fold]) relative to the uninduced level (Fig 1). Treatment with NEM, a thiol-depleting agent, also induced the *nfuA* expression by 13.2 ± 2.6-fold. Nonsignificant induction of the gene expression was observed with H_2_O_2_-treated cells. The results are consistent with the observations from a transcriptome analysis, in which H_2_O_2_ did not induce *nfuA* expression in *P*. *aeruginosa* [11].

Beside oxidant treatments, the level of intracellular iron is a major governing factor in bacterial oxidative stress. Experiments were extended to assess the effects of intracellular iron levels on *nfuA* expression. PAO1 cultures were treated with either 2,2’-dipyridyl, an intracellular iron chelator, or FeCl_3_ to create iron-deplete and iron-excess conditions, respectively. The results revealed no alteration in the *nfuA* expression level under the iron-excess condition, while the iron-deplete condition caused a 3.1 ± 0.6-fold increase in the expression level of *nfuA* compared with the uninduced control (Fig 1).

In summary, the expression of *P*. *aeruginosa nfuA* was highly induced (over 10-fold) in response to PB and NEM treatments and moderately induced (from 3 to 8-fold) by CuOOH, tBOOH, PQ, NaOCl and 2,2’-dipyridyl. The stress-inducible pattern of *nfuA* expression is comparable to that of genes in the *iscR* regulon [12, 13], where the expression is regulated by IscR, a [Fe-S] cluster containing a transcription regulator [6].

### IscR derepresses *nfuA* expression under oxidant exposure

**Fig 2:**
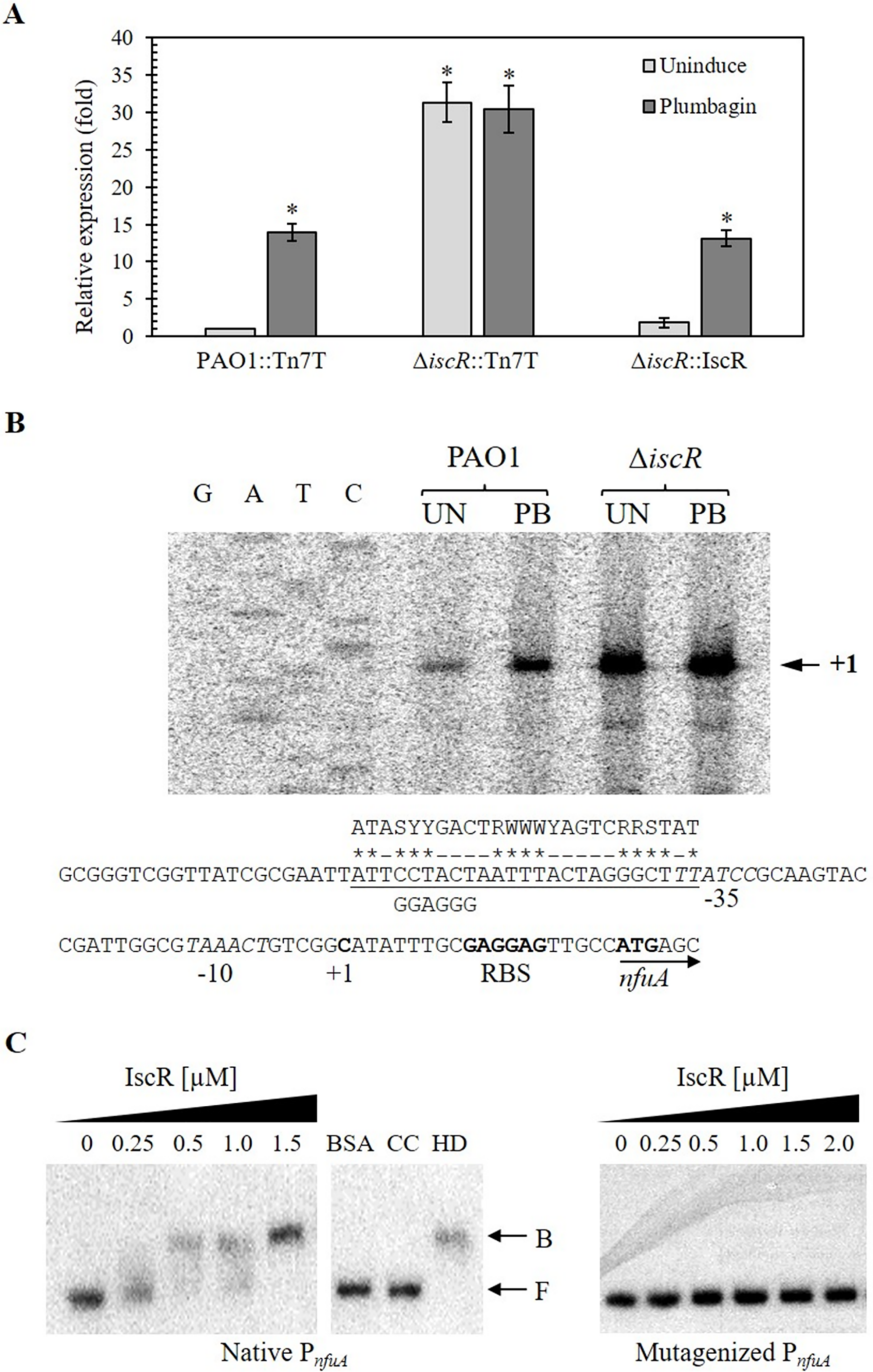
IscR-regulated *nfuA* expression and *nfuA* promoter analysis. (A) IscR-regulated *nfuA* expression. RNA samples were isolated from uninduced and 0.5 mM plumbagin (PB)-induced cultures of the indicated *P*. *aeruginosa* strains. qRT-PCR using primers BT2841 and BT2860 for monitoring *nfuA* expression and performed as described in the Materials and Methods. Relative expression (fold) is defined as the changes in the *nfuA* expression levels across multiple samples relative to the level of the uninduced culture of PAO1. The data are presented as the means ± SD from three independent experiments. (B) The primer extension assay was performed using ^32^P-labeled primer BT3577 and RNA extracted from *P*. *aeruginosa* PAO1 and Δ*iscR* grown under uninduced (UN) and 0.25 mM PB-induced conditions. G, A, T, and C represent the DNA ladder sequence prepared using ^32^P-labeled primer BT3577 and plasmid pP_*nfuA*_ as the template. The arrowhead indicates the transcription start site (+1). The -10 and -35 elements are in italic type. The consensus sequence of the Type-I *E*. *coli* IscR-binding site is aligned above the corresponding underlined sequence, and the homologous bases are marked with asterisks. The mutated IscR-binding site on the *nfuA* promoter was aligned below the underlined sequence line. The putative ribosome-binding site (RBS) is indicated in bold type. (C) The electrophoretic mobility shift assay was performed using ^32^P-labeled native or mutagenized *nfuA* promoter fragments and increasing concentrations of purified IscR. CC and HD represent an addition of 1 μg unlabeled *nfuA* promoter and 2.5 μg of heterologous DNA (pUC18 plasmid), respectively, to the binding reaction mixtures containing 3.0 μM IscR. F and B indicate free and bound probes, respectively.

IscR is a transcriptional regulator regulating several genes implicated in [Fe-S] cluster biogenesis [6, 12, 13]. The pattern of *nfuA* expression in response to stress conditions showed similarity to the expression patterns of IscR-regulated genes [6]. The roles of IscR in the regulation of *nfuA* expression were examined. Treatment with PB, an inducer of IscR, in PAO1 and in an *iscR* mutant [6] showed that PB induced *nfuA* expression in PAO1 (13.9 ± 1.2-fold) but not in the *iscR* mutant, in which *nfuA* was constitutively highly expressed (Fig 2A). The PB induction was restored in the Δ*iscR* complemented strain (Fig 2A, Δ*iscR*::IscR). Moreover, a recent study indicates that copper ions exert their primary toxicity through degradation of [Fe-S] clusters [14]. As IscR contains an [2Fe-2S] cluster, we examined if treatment with copper ions would enhance the *nfuA* expression level. The results showed that treatment with CuCl_2_ at a sublethal concentration (5 mM) somewhat induced the *nfuA* expression (6.6 ± 1.0-fold), while the lower concentration ranging from 100 μM to 2.5 mM failed to induce the *nfuA* expression (data not shown).

Inactivation of *iscR* leads to derepression of the *nfuA* promoter/operator and allows a high level of expression of *nfuA*. IscR acts as a transcriptional repressor of *nfuA* expression. Furthermore, oxidant induction of *nfuA* expression most likely results from oxidant reaction with the [Fe-S] cluster of IscR, leading to an alteration in the repressor-binding ability and derepression of the target promoter/operator. Thus, oxidant-induced expression of *nfuA* in PAO1 is dependent on *iscR*.

### IscR specifically binds to the Type-I IscR-binding motif on the *nfuA* promoter

IscR binds specifically to the consensus sequence located adjacent to the target gene promoter [12, 13]. We experimentally identified the transcription start site (+1) to map the putative promoter region of *nfuA* using a primer extension experiment. Total RNA samples were purified from PAO1 cultures grown in uninduced and PB-induced conditions. Fig 2B demonstrates the primer extension products corresponding to the +1 site located at the C residue 20 nucleotides upstream of the *nfuA* translation initiation codon ATG. The putative *nfuA* promoter -35 and -10 elements were identified as TTATCC and TAAACT, respectively, and were separated by 17 nucleotides. The sequence ATTCCTACTAATTTACTAGGGCTTT, which shares a high score of identity (54%, 14 out of 25) with the consensus sequence for the Type-I *E*. *coli* IscR binding site ATASYYGACTRWWWYAGTCRRSTAT, was identified on the sequence overlapping the -35 motif (Fig 2B). The data suggest that IscR regulates the expression of *nfuA* through binding to its promoter region. The *nfuA* primer extension results from RNA prepared from uninduced and PB-induced samples showed similar size products and indicated that the transcription of *nfuA* under both conditions were driven by the same promoter. The higher amount of the primer extension products as judged from increased band intensity from the PB-induced sample was consistent with the real-time expression analysis in that PB is a potent inducer of *nfuA* expression (Fig 2A). The *nfuA* primer extension analysis of RNA samples prepared from uninduced and PB-induced Δ*iscR* mutants produced similar product sizes and quantities to as well as much higher product band intensities than the uninduced PAO1 sample, supporting constitutive high expression of *nfuA* in the mutant. The results are consistent with RT-PCR analysis of *nfuA* expression in Δ*iscR* (Fig 2A). Moreover, the results from Northern blot analysis hybridized with a *nfuA*-specific probe showed a positive band of 0.6 kb, indicating that *nfuA* is transcribed as a monocistronic mRNA (data not shown).

IscR regulation of its target gene expression has been partly elucidated in *E*. *coli*, *Xanthomonas campestris* and *P*. *aeruginosa* [6, 15–19]. IscR is known to act as either a repressor or an activator of gene expression, depending on the regulator binding to the promoters [6, 12, 13, 16]. IscR functions as a repressor of its own operon, *iscRSUA*, but as an activator of the *sufABCDSE* operon [15, 18]. The latter function requires coordinated regulators, i.e., OxyR, Fur and IHF [15, 18]. Our finding that the expression of *nfuA* was constitutively high in the *iscR* mutant suggests that IscR was a repressor for *nfuA*. This scenario is in line with that reported in *E*. *coli*, where IscR is a transcriptional repressor of *nfuA* [20]. Under physiological conditions, the [Fe-S] ligating IscR binds the *nfuA* promoter and represses its expression. Upon oxidation, disassembly of the [Fe-S] from the IscR leads to derepression of *nfuA* [20]. We showed here that oxidants and iron-depleting conditions are potent inducers of *nfuA* gene expression. Several oxidants are capable of causing oxidative damage, leading to disassembly of the [Fe-S] cluster, thereby deactivating the repressor IscR.

EMSA was conducted using purified *P*. *aeruginosa* IscR protein [6] and a *nfuA* promoter fragment containing the putative IscR binding box. The results showed that purified 6His-tagged IscR was specifically bound to the *nfuA* promoter fragment (Fig 2C). The binding specificity of purified IscR was demonstrated through the addition of excess unlabeled *nfuA* promoter fragment, which competed with the labeled promoter fragment in the IscR-binding complexes (Fig 2C). Furthermore, an unrelated protein (BSA) that was added did not bind to the *nfuA* promoter fragment. The results indicated that IscR binds specifically and directly to the *nfuA* promoter. Site-directed mutagenesis was performed to test if the proposed Type-I IscR-binding site (Fig 2B) functions as the binding site by mutating the putative Type-I IscR-binding site from part of the consensus sequence CCTACT to GGAGGG (Fig 2B). As shown in Fig 2C, increasing levels of purified IscR up to 2.0 mM (greater than the concentration at which purified IscR completely bound the wild-type *nfuA* promoter) was unable to bind the mutated *nfuA* promoter (Fig 2C). The results indicated that the Type-I IscR-binding site was responsible for the binding of IscR to the *nfuA* promoter. As the Type-I IscR-binding site overlapped the -35 promoter motif of the *nfuA* promoter (Fig 2B), binding of IscR would prevent RNA polymerase from binding to the promoter and thereby inhibiting the transcription of *nfuA*. These results support the role of IscR as a transcriptional repressor of *nfuA* gene expression. However, the requirement of [Fe-S] cluster for the binding of IscR to the *nfuA* promoter is under investigation.

### The effects of *nfuA* inactivation on either [2Fe-2S] or [4Fe-4S] containing protein functions

**Fig 3:**
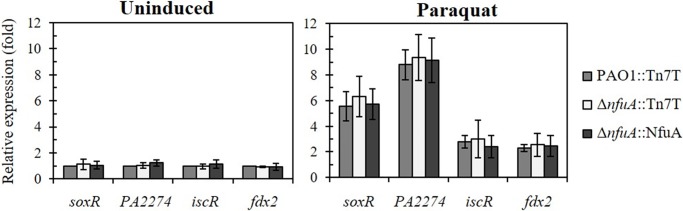
Expression levels of *soxR*, *iscR* and their targeted genes in *P*. *aeruginosa* strains. Analysis of the expression levels of genes encoding [2Fe-2S]-containing transcriptional regulators, namely, SoxR and IscR, and their targeted genes, *soxR*, *PA2274*, *iscR* and *fdx2*, in *P*. *aeruginosa* wild type (PAO1::Tn7T), **Δ***nfuA* mutant (**Δ***nfuA*::Tn7T) and the complemented strain (**Δ***nfuA*::NfuA) grown in uninduced and 0.5 mM Paraquat-induced conditions. qRT-PCR was performed as described in the Materials and Methods, and the data are shown as the fold expression relative to the level of the uninduced PAO1 (PAO1::Tn7T).

NfuA has been reported as an atypical [Fe-S] carrier contributing in part to the maturation of [Fe-S]-containing proteins such as aconitase (AcnB) and the complex I subunit NuoG dehydrogenase [9]. In *P*. *aeruginosa*, the role of NfuA is not known; thus, we investigated the role of NfuA in the functions of [2Fe-2S] and [4Fe-4S]-containing proteins *in vivo*. First, we tested whether inactivation of *nfuA* affects the regulatory function of the characterized [2Fe-2S]-containing transcriptional regulators, SoxR and IscR [16, 21]. Both regulators have a [2Fe-2S] cluster as a functional prosthetic group, and the holo-proteins ([2Fe-2S]-SoxR and [2Fe-2S]-IscR) function as the transcriptional repressor of their target genes [20]. Oxidation of [2Fe-2S] of SoxR and destabilization of [2Fe-2S] of IscR upon exposure to oxidants such as superoxide generators leads to activation or depression of the target genes [22]. If NfuA participates in maturation of these proteins, we would expect alterations in the expression patterns of SoxR- and IscR-regulated genes in response to oxidants. The results in Fig 3 show that deletion of *nfuA* did not affect the uninduced and the PQ-inducible expression profiles of a SoxR-regulated gene (*PA2274* and *soxR*) and an IscR-regulated gene (*fdx2* and *iscR)* compared to profiles obtained in the PAO1 wild type. The results suggest that *nfuA* is not involved in the maturation of the tested [2Fe-2S]-containing proteins.

**Fig 4:**
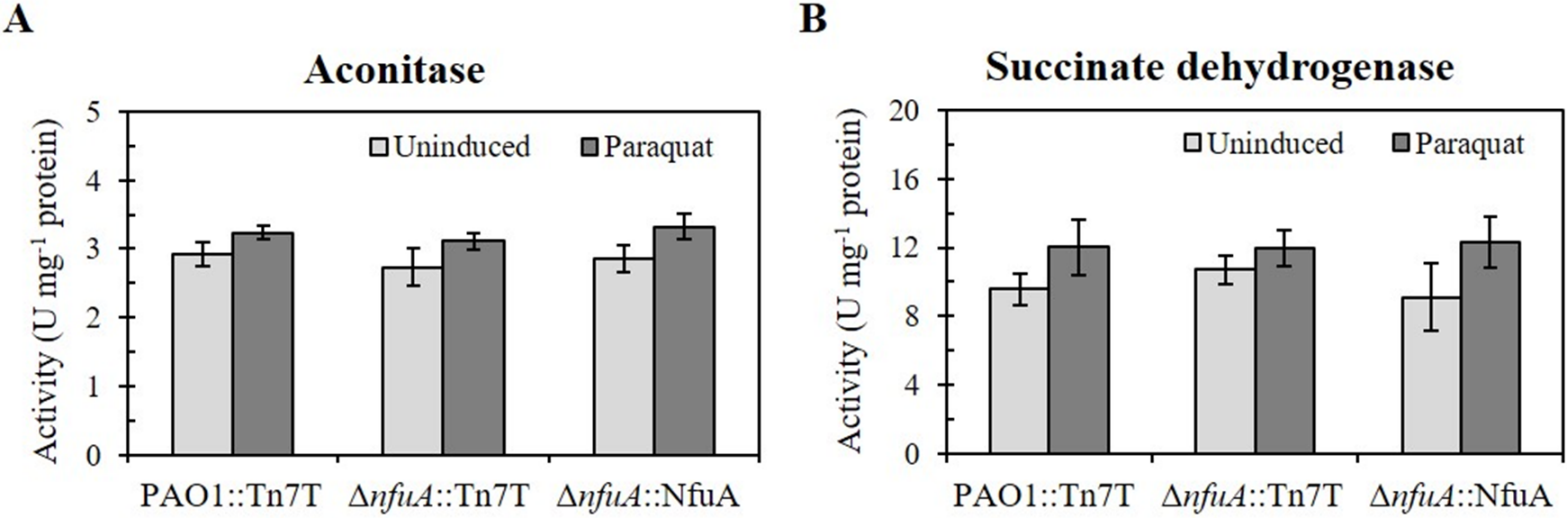
Determination of the activity of iron-sulfur cluster-containing enzymes in *P*. *aeruginosa* strains. The enzyme activity of aconitase (A) and succinate dehydrogenase (B) was measured in the PAO1 and Δ*nfuA* mutant carrying the empty Tn7T vector (PAO1::Tn7T and Δ*nfuA*::Tn7T) as well as in the Δ*nfuA* mutant expressing wild-type NfuA or mutated NfuA (NfuA_C152S_, NfuA_C155S_, and NfuA_C43,47S_) under uninduced and Paraquat-induced conditions as described in the Materials and Methods. The data shown are the means ± SD from three independent experiments.

NfuA is known to function in [4Fe-4S] ligation and transfer to target proteins [2]. Next, the activities of two [4Fe-4S] clusters containing enzymes at their active sites, i.e., aconitase (Acn) and succinate dehydrogenase (Sdh), were monitored in the Δ*nfuA* mutant and in a PAO1 wild type. The Acn and Sdh activities in the Δ*nfuA* mutant were not significantly different from those in the PAO1 wild type under uninduced and paraquat-induced conditions (Fig 4A and 4B). By contrast, in *E*. *coli*, a decrease in aconitase activity has been observed in the Δ*nfuA* mutant [9]. *P*. *aeruginosa* NfuA plays no role in the maturation and transfer of [4Fe-4S] clusters to Acn and Sdh. This finding is in good agreement with the proposed role of NfuA, namely, that it is involved in the ligation and transfer of [Fe-S] clusters only to a subset of target proteins.

### The role of *nfuA* in stress protection

**Fig 5:**
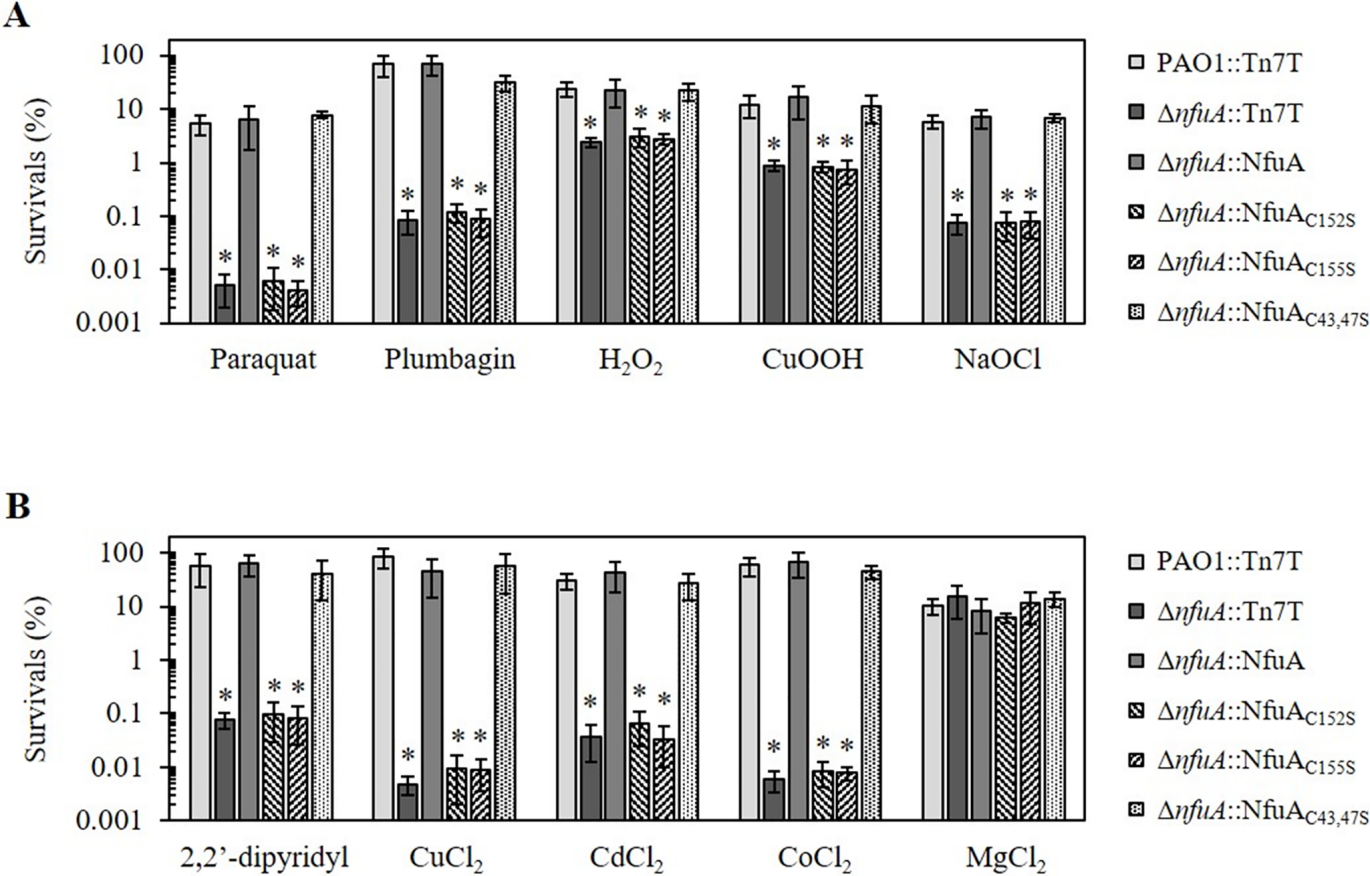
Determination of the resistance levels against oxidative and metal stresses in *P*. *aeruginosa* strains. The resistance levels of PAO1::Tn7T, Δ*nfuA*::Tn7T and the Δ*nfuA*::NfuA mutant strains expressing transposed wild-type NfuA or mutated NfuA (NfuA_C152S_, NfuA_C155S_, and NfuA_C43,47S_) against substances were determined using plate sensitivity assays on plates containing oxidants (A) i.e., 250 μM Paraquat, 1 mM Plumbagin, 0.5 mM H_2_O_2_, 1.6 mM CuOOH, and 0.045% NaOCl, and metals (B) i.e., 1.2 mM 2,2’-dipyridyl, 4.2 mM CuCl_2_, 0.8 mM CdCl_2_, 0.5 mM CoCl_2_, and 5 mM MgCl_2_. Survival (%) was defined as the percentage of colony-forming units (CFU) on plates containing oxidants over the number of CFU on plates without oxidants. The data shown are the means ± SD from three independent experiments. The asterisk indicates statistical significance (paired *t*-test, *p* < 0.05) compared with PAO1 treated with the same condition.

[Fe-S] clusters are known targets for reactions with oxidants. In several organisms, genes involved in [Fe-S] biogenesis are also involved in processes leading to oxidative stress resistance [6, 13, 19, 23–26]. We have reported that deletion of *nfuA* renders *P*. *aeruginosa* more susceptible to fluoroquinolone antibiotics [10]. To further evaluate the role of *P*. *aeruginosa nfuA* in processes of oxidative stress resistance, the level of resistance against various oxidants in the Δ*nfuA* mutant was determined and compared with that of the PAO1 wild type. The Δ*nfuA* mutant was extremely sensitive to superoxide generators, showing 10^4^-fold and 10^3^-fold increased sensitivity to PQ and PB, respectively, compared to the PAO1 wild type (Fig 5A). The sensitivities of the Δ*nfuA* mutant against peroxides, both H_2_O_2_ and CuOOH, and NaOCl were also increased 10-fold relative to the wild-type level. The altered phenotypes observed in the Δ*nfuA* mutant could be complemented by a single copy of *nfuA* that was transposed into the mutant chromosome using a mini-Tn7 vector (Fig 5A). The Δ*nfuA* mutant showed increased sensitivity to a variety of Reactive Oxygen Species, ROS and, particularly, to superoxide generators. [Fe-S] clusters in enzymes and proteins are proven targets for ROS [3, 14, 27].

Next, we tested if *nfuA* has physiological roles in protecting bacteria from an iron-depleting condition. The Δ*nfuA* mutant showed 10^3^-fold increased sensitivity to the intracellular iron chelator, 2,2’-dipyridyl, relative to that of the wild type. The phenotype suggests that there is less free intracellular Fe^2+^ available in the *nfuA* mutant. In addition, the level of intracellular free Fe^2+^ in the Δ*nfuA* mutant was indirectly determined using a streptonigrin sensitivity assay [28]. Streptonigrin is a redox cycling antibiotic that generates free radicals, and its bactericidal activity is enhanced by metal ions, notably Fe^2+^. The levels of free Fe^2+^ directly correlate with the bactericidal activity of the antibiotic [28]. Both the *nfuA* mutant and PAO1 showed the same survival rate of approximately 1% after treatment with 50 μg ml^-1^ streptonigrin for 2 h (data not shown), suggesting that the basal levels of free Fe^2+^ in the cytoplasm of the *nfuA* mutant and PAO1 wild type were similar. Thus, the defective [Fe-S] cluster transfer and assembly in the *nfuA* mutant does not lead to an increase and is more likely to cause a minor reduction in free intracellular ferrous iron. The increased susceptibility of the *nfuA* mutant to an iron chelator was probably due to defects in [Fe-S] assembly and transfer processes leading to a lowering of the total intracellular free iron pool. A similar phenotype has been observed in *E*. *coli*, where *nfuA* is shown to be important under iron-deprivation conditions [7].

Metal toxicities in bacteria have been linked to the ability of metals to either directly react with or replace various metal-containing proteins, resulting in induced oxidative stress [29–32]. Hence, the role of *nfuA* in conferring resistance against metal toxicities using a plate sensitivity assay was evaluated. The results in Fig 5B show that the *nfuA* mutant was more sensitive to copper (CuCl_2_, 10^4^-fold), cadmium (CdCl_2_, 10^3^-fold) and cobalt (CoCl_2_, 10^3^-fold) than the PAO1 wild type. The Δ*nfuA* mutant metal-hypersensitive phenotypes were restored in the complemented strain (Fig 5B). Moreover, no alterations in the levels of resistance of the Δ*nfuA* mutant to the nonredox metal Mg were observed.

Redox metals, including Cu^2+^, Cd^2+^, and Co^2+^, and oxidants could exert their toxicities through the degradation of [4Fe-4S] clusters, resulting in a damaged cluster and release of Fe^2+^. The ferrous ion could participate in the Fenton reaction and generate highly reactive hydroxyl radicals [3, 14, 33]. NfuA has functions as a [Fe-S] cluster carrier and transfers the cluster to a subset of target proteins. In the *nfuA* mutant, this process would lead to a reduction in the number of functional [Fe-S] clusters transferred to target proteins and subsequent loss of enzymatic function, resulting in hypersensitive phenotypes to oxidants and redox metals. The data indicate that NfuA has important functions in [Fe-S] cluster ligation and transfer under stress conditions. Moreover, NfuA likely plays some roles in intracellular iron homeostasis that could be important under iron deprivation conditions.

### Mutagenesis and functional analysis of conserved cysteine residues

*P*. *aeruginosa* NfuA contains two conserved motifs: C_43_XXXC_47_ of the degenerate ATC domain and C_152_XXC_155_ of the Nfu domain. Experiments were designed to examine the functionality of these two conserved domains in protecting PAO1 from stress. Site-directed mutagenesis changing C43S and C47S (C43,47S) of the ATC domain, C152S and C155S of the Nfu domains was performed to test the functionality of these motifs of the NfuA protein in protecting bacteria against stress. The mutated *nfuA* genes were cloned into a mini-Tn7 vector, and the recombinant clones were transposed into the *P*. *aeruginosa* Δ*nfuA* mutant. Expression of NfuA_C43,47S_ complemented the phenotypes of increased sensitivity to oxidative stress (PQ, PB, H_2_O_2_, CuOOH and NaOCl), metal stress (CuCl_2_, CdCl_2_, CoCl_3_) and iron depletion (2,2’-dipyridyl) in the Δ*nfuA* mutant to levels similar to those attained by a parental strain (Fig 5A and 5B). By contrast, the expression of mutated *nfuA* that had either conserved cysteine residues C152 or C155 of the C_152_XXC_155_ motif of the Nfu domain converted to serine failed to complement the phenotypes of increased sensitivity to oxidative stress, metals and iron depletion in the Δ*nfuA* mutant (Fig 5A and 5B). Expression and Western blot analyses were performed to ensure that mutations of the *nfuA* gene (C152S, C155S, and C43,47S) did not affect expression of the genes at both transcriptional and translational levels (S1 Fig). The deletion analysis of NfuA indicates that NfuA domain is primarily essential for the protein functions. The ATC domain is thought to function in recognizing the target proteins and promote [Fe-S] cluster transferring processes [7, 9]. Our results showed that mutation at the C43S and C47S residues in the C_43_XXXC_47_ motif of the ATC type domain had no effects on the ability of mutated *nfuA* to complement the stress-hypersensitive phenotypes of the mutants, suggesting that the target protein recognition functions of the motif under stress conditions do not have primarily important roles for protecting the Δ*nfuA* mutant from lethal levels of oxidative stress, metals and iron-depletion conditions. The CXXC motif of Nfu domain is thought to be involved in the ligation and binding of [4Fe-4S] clusters and to facilitate transfer of the clusters to target proteins [9]. [Fe-S] clusters are targets involved in reactions with oxidants and reactive metals. The clusters are easily damaged by these stresses, which lead to decrease functionality or inactivation of these [Fe-S] containing proteins and result in increased sensitivity to lethal levels of these stresses. Thus, NfuA has ability to assist the bacteria in maintaining a balance between the transfer rates of newly formed clusters to replace stress-damaged clusters. This balance is likely important for the functionality of [Fe-S]-containing proteins involved in stress-protective mechanisms, which is reflected by the overall resistance levels to stress. As shown herein, mutations in the Nfu domain hinder the binding and transfer of [Fe-S] clusters to stress-damaged [Fe-S] clusters and prevent NfuA from complementing the stress hypersensitive phenotypes of the Δ*nfuA* mutant. The complementation of the stress hypersensitive phenotypes even suggest that NfuA could function as a chaperone and/or in the repair of stress damaged [Fe-S] clusters. Similar, results observed in *E*. *coli* have shown that mutations of a cysteine residue in the CXXC motif of the Nfu-type domain abolishes its ability to complement the stress hypersensitive phenotypes of *nfuA* mutants [7].

### *nfuA* is required for proper growth under anaerobic condition

**Fig 6:**
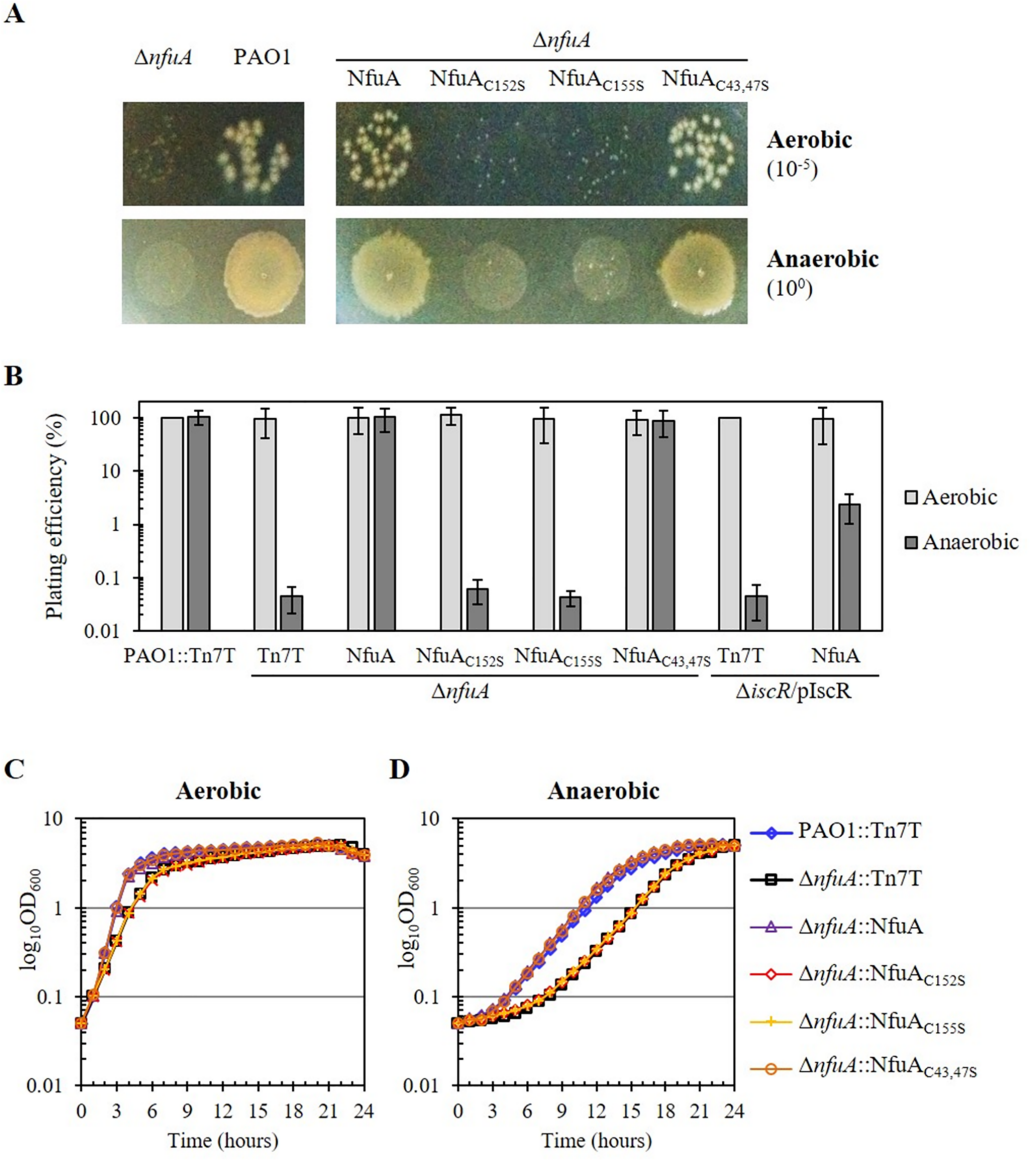
*P*. *aeruginosa* and Δ*nfuA* mutant plating efficiency and growth under aerobic and anaerobic conditions. The plating efficiency and growth of PAO1 and various mutant strains were determined. Bacteria were grown in LB for aerobic conditions and in LB plus 1% KNO_3_ for anaerobic conditions. The plating efficiency was defined as the number of CFU under anaerobic conditions divided by the number of CFU under aerobic conditions. The asterisk indicates statistically significant differences (paired *t*-test, *p* < 0.05) compared with the aerobic condition. (A) The plating efficiency and colony morphology of PAO1 and **Δ***nfuA* mutant strains under aerobic and anaerobic conditions was determined. The PAO1, **Δ***nfuA*::NfuA strains expressing wild-type NfuA or mutated NfuA (NfuA_C152S_, NfuA_C155S_, and NfuA_C43,47S_) were spotted onto plates containing 1% KNO_3_ and incubated under aerobic and anaerobic conditions. (B) Plating efficiency of the PAO1 (PAO1::Tn7T), **Δ***nfuA* mutant (**Δ***nfuA*::Tn7T) and various complemented strains as in (A), with the addition of **Δ***iscR*/pIscR and **Δ***iscR*::NfuA/pIscR strains performed under aerobic and anaerobic conditions. Growth of *P*. *aeruginosa* strains under aerobic (C) and anaerobic (D) conditions in broth supplemented with 1% KNO_3_ incubated at 37°C with 180 rpm shaking was determined. The OD_600nm_ was monitored at hourly intervals for 24 h. Typically representative results of five independent experiments are shown.

We observed that colonies on the LB agar plate of the Δ*nfuA* mutant grown under an aerobic atmosphere were smaller than those of the PAO1 wild type (Fig 6A). However, the plating efficiencies of the PAO1 wild type and Δ*nfuA* mutant on LB plates incubated under aerobic conditions did not differ (Fig 6A and 6B). Nonetheless, the aerobic growth of the Δ*nfuA* mutant in LB broth showed a small growth defect compared to the growth of the PAO1 wild type (Fig 6C), suggesting that *nfuA* is required for protecting bacteria against toxic metabolites generating oxidative stress from normal aerobic growth of *P*. *aeruginosa*. Moreover, the growth of the Δ*nfuA* mutant under an anaerobic atmosphere was monitored on both LB agar plates and broth. Under anaerobic condition, PAO1 was unable to grow on LB plated with or without glucose supplementation (data not shown). Thus, the medium was supplemented with nitrate to allow anaerobic growth of *P*. *aeruginosa* using nitrate respiration [34]. Unexpectedly, the Δ*nfuA* mutant exhibited a severe reduction of more than 10^3^-fold in the plating efficiency relative to that of the PAO1 wild type (Fig 6A and 6B). Furthermore, the doubling time under anaerobic growth of the Δ*nfuA* mutant was considerably increased to 134 min compared to 119 min for PAO1 (Fig 6D). The reduced plating efficiency and growth retardation phenotypes of the Δ*nfuA* mutant was fully restored in the complemented strain (Δ*nfuA*::NfuA). However, the expression of the mutated *nfuA* genes in which conserved cysteine residues in the Nfu-type domain were mutated (either NfuA_C152S_ or NfuA_C155S_) were unable to complement the growth defect phenotypes under both aerobic and anaerobic conditions. The expression of mutated *nfuA* in the ATC domain (NfuA_C43,47S_) complemented the growth retardation phenotypes to levels similar to those of wild-type NfuA (Fig 6A, 6B, 6C and 6D). The results illustrated the importance of *nfuA* to *P*. *aeruginosa* growth, especially under anaerobic conditions, and the involvement of the Nfu domain in binding and transferring [4Fe-4S] clusters to require enzymes for anaerobic growth. To our knowledge, this is the first report showing an essential role of *nfuA* for anaerobic growth in bacteria.

In PAO1, overexpression of *iscR* results in an anaerobic growth defect [6]. Here, we showed that IscR was a transcriptional repressor of *nfuA*. Hence, high levels of IscR lead to low *nfuA* expression in the IscR-overexpressing strain, which may be responsible for the observed anaerobic growth defect phenotype. An experiment was performed to test the assumption by expressing *nfuA* in an *iscR-*overexpressing strain and measuring the anaerobic growth. The results (Fig 6B) showed that increased expression of *nfuA* from the *lacZ* promoter (in strain Δ*iscR*::NfuA/pIscR) partially restored the anaerobic growth defect phenotype of the *iscR-*overexpressing strain to a level 10^2^-fold higher than that of the *iscR-*overexpressing strain but just over 10-fold lower than the PAO1 level. These results confirm that low levels of *nfuA* expression in *iscR-*overexpressing strains contribute to the anaerobic-growth-defect phenotype of the strain.

Experiments were extended to determine the *nfuA* expression levels in anaerobic cultures in PAO1. One percent KNO_3_ was added into the culture medium to allow anaerobic growth of *P*. *aeruginosa* through nitrate respiration; we also tested the effect of nitrate supplementation on *nfuA* expression. The results showed that the presence of nitrate (LB + KNO_3_) induced a 2.2 ± 0.8-fold increase in *nfuA* expression under aerobic conditions compared to the expression in PAO1 grown aerobically in LB medium (Fig 1). The expression of *nfuA* in PAO1 grown in LB+KNO_3_ under anaerobic conditions was further induced (2.6-fold) compared to the expression in aerobically grown cells in a similar medium (Fig 1). Thus, the *nfuA* expression was also induced in response to the presence of 1% nitrate and in anaerobic conditions. This finding illustrates the important correlation between gene expression profiles and biochemical and physiological functions in the nitrate respiration under anaerobic conditions. Moreover, we found that addition of 1% nitrate could significantly increase *nfuA* expression in the Δ*iscR* mutant grown under aerobic condition suggesting that nitrate-induced the expression of *nfuA* is not mediated by IscR (data not shown). Thus, the mechanism by which nitrate induces *nfuA* expression is unknown.

**Fig 7:**
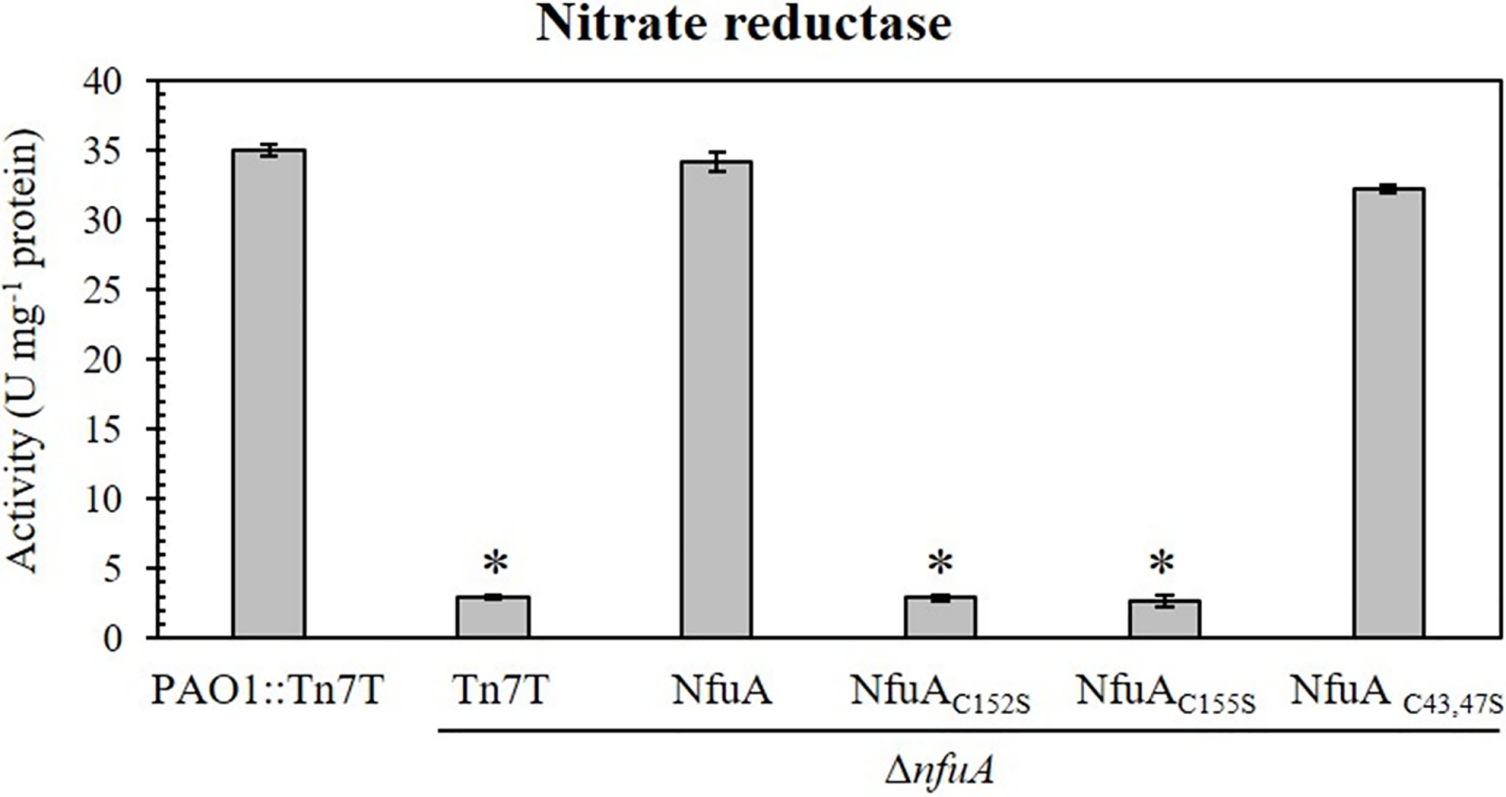
Determination of nitrate activity in *P*. *aeruginosa* strains. Nitrate reductase activity was measured in the PAO1 and Δ*nfuA* mutant carrying Tn7T vector (PAO1::Tn7T and Δ*nfuA*::Tn7T), and the Δ*nfuA* mutant expressing wild-type NfuA or mutated NfuA (NfuA_C152S_, NfuA_C155S_, and NfuA_C43,47S_), as described in the Materials and Methods. The data shown are the means ± SD from three independent experiments.

*P*. *aeruginosa* has a set of denitrification enzymes capable of reducing nitrate to molecular nitrogen, and these enzymes allow the bacterium to grow under anaerobic conditions in the presence of nitrate. As the Δ*nfuA* mutant exhibited a severe growth defect under anaerobic conditions, we postulated that NfuA may be needed for the denitrification process. Nitrate reductases contain [Fe-S] cluster prosthetic groups. The total nitrate reductase activity in the Δ*nfuA* mutant grown under anaerobic conditions was determined. The total nitrate reductase activity level in the Δ*nfuA* mutant (2.9 ± 0.2 U mg^-1^ protein) was significantly lower than that in the PAO1 wild type (35.0 ± 0.4 U mg^-1^ protein), and the activity could be restored to the wild-type level in the complemented strain Δ*nfuA*::NfuA (34.2 ± 0.7 U mg^-1^ protein) (Fig 7). The results suggest that NfuA is required for full activity of nitrate reductases, the enzymes that play a crucial role in maintaining proper growth under anaerobic conditions. However, direct involvement of NfuA in [Fe-S] delivery to nitrate reductase needs further investigation.

Furthermore, the NfuA domain that is responsible for the NfuA effect on nitrate reductase enzyme activity was determined. The Δ*nfuA* mutant expressed mutant NfuA proteins (NfuA_C152S_, NfuA_C155S_ and NfuA_C43, 47S_). As shown in Fig 7, the expression of NfuA_C152S_ and NfuA_C155S_ could not complement the reduced total nitrate reductase activity of the Δ*nfuA* mutant, indicating that the Nfu domain is critical for the NfuA effect on the activity of nitrate reductases. Mutation of C43 and C47 on the ATC domain (NfuA_C43,47S_) caused no adverse effect on the protein ability to complement the nitrate reductase activity of the Δ*nfuA* mutant (Fig 7).

More recently, it has been proposed in *E*. *coli* that NfuA assists ErpA in delivery of [Fe-S] cluster to the target proteins under unfavorable conditions such as oxidative stress [35]. High expression of *erpA* could complement the paraquat-sensitive phenotype of *E*. *coli nfuA* mutant [35]. Nevertheless, expression of a PAO1 *erpA*-homolog from pErpA_pa_ failed to restore the paraquat-sensitive phenotype of the Δ*nfuA* mutant (data not shown). Thus, *P*. *aeruginosa* NfuA may function differently from *E*. *coli* NfuA.

### *nfuA* mutant has attenuated virulence in the *Caenorhabditis elegans* model host-pathogen system

**Fig 8:**
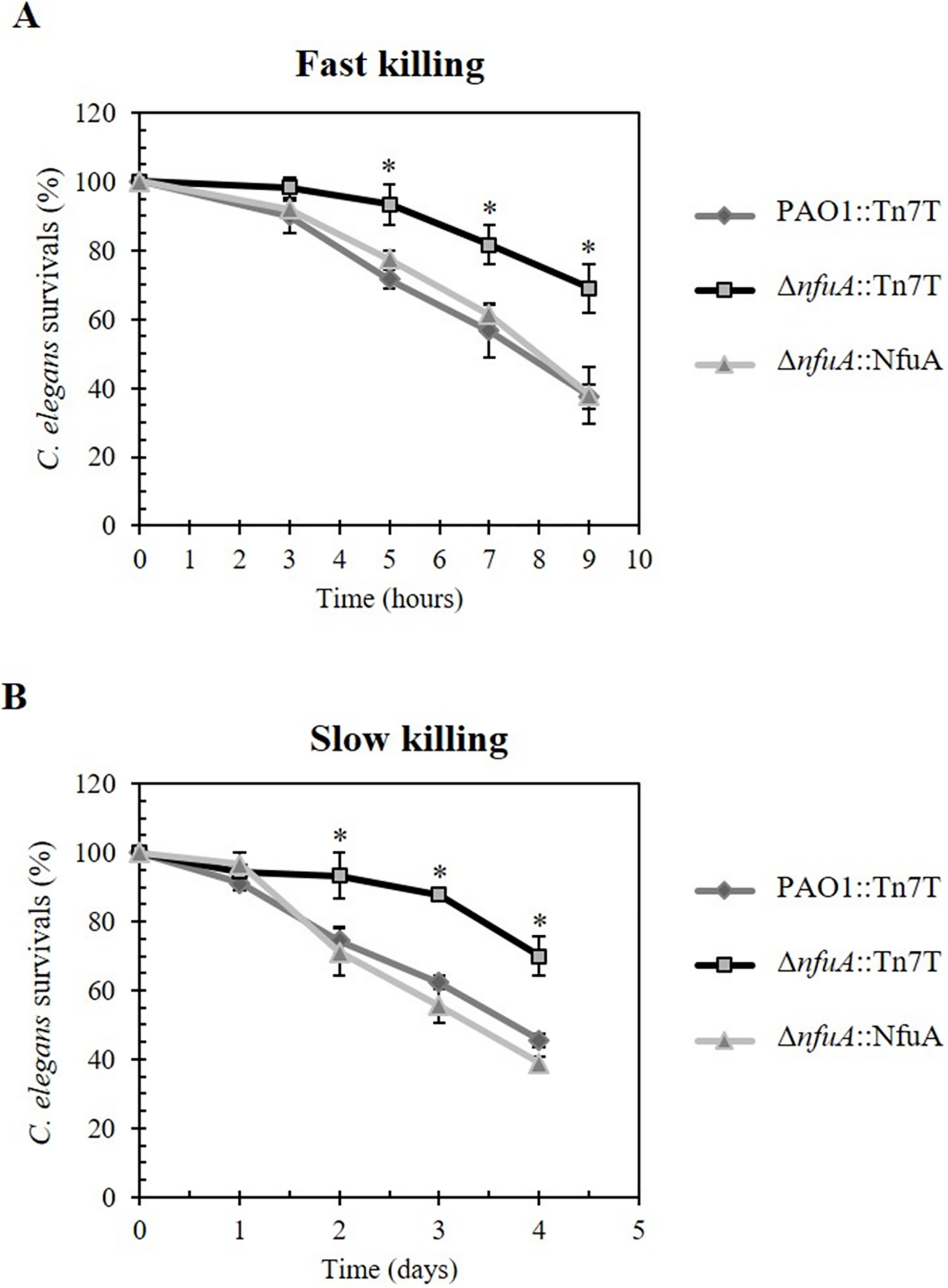
*P*. *aeruginosa* virulence test using the *Caenorhabditis elegans* model host model. Fast (A) and slow (B) killing assays were performed to determine the virulence of *P*. *aeruginosa* PAO1::Tn7T, **Δ***nfuA*::Tn7T and the complemented strain (**Δ***nfuA*::NfuA) as described in the Materials and Methods. Live worms were scored at 3, 5, 7 and 9 h for fast-killing experiments and at 1, 2, 3 and 4 days for slow-killing experiments. The data were analyzed from nine biological replicates, and the means ± SD are shown.

The Δ*nfuA* mutant showed increased sensitivity to stresses and growth defects, and thus, we tested whether loss of *nfuA* function affects bacterial pathogenicity using a *C*. *elegans* model host system. *C*. *elegans* has been proposed as a model for studying host-pathogen interactions due to the susceptibility of *C*. *elegans* to different virulent phenotypes of *P*. *aeruginosa* [36, 37]. Fast and slow killing assays of *P*. *aeruginosa* towards *C*. *elegans* were performed. Fast killing is mediated by diffusible toxins released from *P*. *aeruginosa* and does not need live bacteria to kill the worms, whereas slow killing requires bacterial colonization in the worm gut to exhibit virulence [36]. The Δ*nfuA* mutant was attenuated for virulence by showing a roughly two-fold reduction in its ability to kill *C*. *elegans* compared to the PAO1 wild type in both fast and slow killing assays (Fig 8A and 8B). The phenotypes were restored in the complemented mutant (Δ*nfuA*::NfuA) strain.

Thus, *nfuA* contributes to the virulence of *P*. *aeruginosa*. Defects in the nitrate dissimilation pathway have been shown to cause attenuation of the virulence phenotype and altered biofilm formation of *P*. *aeruginosa* [38]. We speculate, therefore, that the virulence attenuation in the Δ*nfuA* mutant is at least in part due to lowered nitrate reductase activity and anaerobic growth retardation. Physiologically, the ability of *P*. *aeruginosa* to grow in anaerobic conditions is also important for chronic infection in the cystic fibrosis lung, in which the bacteria must face an anaerobic microenvironment [39].

## Materials and methods

### Bacterial strains and growth conditions

All *Pseudomonas aeruginosa* strains were raised, maintained and experiments were conducted following procedures approved by the Committee of Biosafety, Faculty of Science, Mahidol University (MUSC2017-001).

All bacterial strains and plasmids used in this study are listed in Table 1. *Pseudomonas aeruginosa* (PAO1) strains were grown aerobically in the Luria-Bertani (LB) medium at 37°C with shaking at 180 rpm. As required, the medium were supplemented with 200 μg ml^−1^ carbenicillin (Cb), 25 μg ml^−1^ chloramphenicol (Cm) or 30 μg ml^−1^ gentamicin (Gm). To produce more synchronous growth, an overnight culture was inoculated into fresh LB medium to give an optical density at 600 nm (OD_600nm_) of 0.1. Exponential phase cells (OD_600nm_ of about 0.6, after 3 h of growth) were used in all experiments.

**Table 1:** Bacterial strains and plasmids used in this study.

Bacterial strain	Genotype or characteristic	Source
PAO1	wild type	ATCC15692
PAO1::Tn7T	PAO1 transposed with pUC18-Mini-Tn7T-Gm-LAC	In this study
Δ*nfuA*	PAO1 Δ*nfuA* mutant	[10]
Δ*nfuA*::Tn7T	Δ*nfuA* transposed with pUC18-Mini-Tn7T-Gm-LAC	In this study
Δ*nfuA*::NfuA	Δ*nfuA* transposed with pTn7T-NfuA	In this study
Δ*nfuA*::NfuA_C152S_	Δ*nfuA* transposed with pTn7T-NfuA_C152S_	In this study
Δ*nfuA*::NfuA_C155S_	Δ*nfuA* transposed with pTn7T-NfuA_C155S_	In this study
Δ*nfuA*::NfuA_C43,47S_	Δ*nfuA* transposed with pTn7T-NfuA_C43,47S_	In this study
Δ*iscR*	PAO1 Δ*iscR* mutant	[6]
Δ*iscR*/pBBR	Δ*iscR* harboring pBBR1MCS-4	[6]
Δ*iscR*/pIscR	Δ*iscR* harboring pBBR1MCS-4 with full-length *iscR*	[6]
Δ*iscR*::NfuA/pIscR	Δ*iscR* transposed with pTn7T-NfuA harboring pBBR *iscR*	In this study

### Oxidant- and metal-induction experiments

PAO1 and mutant strains were subcultured from overnight cultures to yield an OD_600nm_ of 0.1 and grown for 3 h. For induced cultures, either oxidants or metals were added to the cultures to the indicated concentrations, and the cultures were further grown for 15 min. Uninduced and induced cultures were harvested for RNA extractions and enzyme assays.

### Site-directed mutagenesis of NfuA

PCR-based site-directed mutagenesis was performed as previously described [40] to replace the conserved cysteines (C43, C47, C152, and C155) of NfuA with serine residues. The mutagenic forward and reverse primers used to produce the mutant NfuA were BT3169 and BT3170 for NfuA_C43,47S_; BT2961 and BT2962 for NfuA_C152S_; and BT3238 and BT3239 for NfuA_C155S_. Two-step PCR amplification reactions were conducted using plasmid pNfuA [10] as the template and specific mutagenic primers. The PCR products were digested with Ecl136II and BamHI prior to cloning into pUC18-Mini-Tn7T-Gm-LAC [41] cut with the same enzymes. The resultant plasmids pTn7T-NfuA_C43,47S_, pTn7T-NfuA_C152S_ and pTn7T-NfuA_C155S_ expressing mutant NfuA proteins, NfuA_C43,47S_, NfuA_C152S_ and NfuA_C155S_, respectively, were transposed into the Δ*nfuA* mutant. The accuracy of mutagenesis was verified by DNA sequencing.

### Molecular techniques

General molecular techniques including DNA and RNA preparation, and bacterial transformation were performed using standard protocols [42]. The oligonucleotide primers used in this study are listed in Table 2.

**Table 2:** List of primers used in this study.

Number	Sequence (5’→3’)	Purpose
BT3291	CGAGACGAAGAGGTCGTT	Reverse primer for primer extension of *nfuA*
BT2879	AACCGCTACGAGAACCTC	Forward primer for full length *nfuA*
BT3169	CCTCCATCGCCTACTCCAAGCCGGGCGAGG	Forward primer for mutagenesis of NfuA_C43,47S_
BT3170	TGGAGTAGGCGATGGAGGTTTCCGCGTAC	Reverse primer for mutagenesis of NfuA_C43,47S_
BT2961	GCGGCGGTTCTCAGGGCTG	Forward primer for mutagenesis of NfuA_C152S_
BT2962	CAGCCCTGAGAACCGCCGC	Reverse primer for mutagenesis of NfuA_C152S_
BT3238	CAGGGCAGCGGGATGGTCGAC	Forward primer for mutagenesis of NfuA_C155S_
BT3239	TCCCGCTGCCCTGACAACCG	Reverse primer for mutagenesis of NfuA_C155S_
BT4099	ATTGGAGGGAATTTACTAGGGCTTTATCCG	Forward primer for mutagenesis of *nfuA* promoter
BT4100	ATTCCCTCCAATAATTCGCGATAACCGACC	Reverse primer for mutagenesis of *nfuA* promoter
BT2781	GCCCGCACAAGCGGTGGA	Forward primer for qRT-PCR of *16S rRNA*
BT2782	ACGTCATCCCCACCTTCC	Reverse primer for qRT-PCR of *16S rRNA*
BT2841	ACCATCCCGCAGCCCTG	Reverse primer for qRT-PCR of *nfuA*
BT2860	ACCGCCATCGCCCTGAAG	Forward primer for qRT-PCR of *nfuA*
BT3186	TATCTCCGAACGCCAAGG	Forward primer for qRT-PCR of *iscR*
BT3187	GGTGGTGGGTCAGACAGG	Reverse primer for qRT-PCR of *iscR*
BT3555	CGCAATGGCATCGAGATCGA	Forward primer for qRT-PCR of *fdx2*
BT3556	GATAGCCGCGAATCGGGCTC	Reverse primer for qRT-PCR of *fdx2*
BT3046	CCAGCGGGTCGGCATTCC	Forward primer for qRT-PCR of *soxR*
BT3047	AGGCCTGGAGCGACAGGC	Reverse primer for qRT-PCR of *soxR*
BT3351	ACCCGCCAGCCAGTTGTC	Forward primer for qRT-PCR of *PA2274*
BT3352	CACGCTTTTCGCCCCCAG	Reverse primer for qRT-PCR of *PA2274*
Tn7S	GATGGGAACTGGGTGTAGCG	Reverse primer on pUC-Mini-Tn7T-Gm-LAC

### Construction of pTn7T-*nfuA* for a single copy complementation of the Δ*nfuA* mutant

A full-length *nfuA* excised from of pNfuA plasmid [10] digested with EcoRV and BamHI was cloned into the Ecl136II- and BamHI-cut pUC18-Mini-Tn7T-Gm-LAC [41] to generate pTn7T-NfuA. The recombinant plasmid was transposed into the Δ*nfuA* mutant, yielding a complemented mutant strain (Δ*nfuA*::NfuA), in which *nfuA* was expressed under *lac* promoter. Confirmation of transposition was performed as described [41].

### Primer extension

Primer extension experiments were performed as previously described [6] using 10 μg total RNA, ^32^P- labeled primer BT3291, 200 U Superscript II RNaseH minus M-MLV reverse transcriptase (Life Technologies, USA) and incubated at 42°C for 60 min. The primer extension products were analyzed on a sequencing gel (8% polyacrylamide-7 M urea). A DNA ladder was generated using fmol DNA cycle sequencing system (Promega). The template for the sequencing ladder was an *nfuA* promoter fragment and the primer was ^32^P- labeled primer BT3291.

### Site-directed mutagenesis on *nfuA* promoter

The putative IscR binding site 5’ATTCCTACTAATTTACTAGGGCTT3’ situated on the *nfuA* promoter was converted to 5’ATTGGAGGGAATTTACTAGGGCTT3’ using a mutagenic primer pair BT4099 and BT4100 and pP_*nfuA*_ (pBBR1MCS-4 [43] containing putative *nfuA* promoter fragment amplified from PAO1 genomic DNA using primers BT2879 and BT3291) as DNA template. The mutagenized promoter was cloned into pBBR1MCS-4 to generated pP*_nfuA_ and verified by DNA sequencing.

### Gel mobility shift assay

6His-tagged IscR protein from *P*. *aeruginosa* was purified using the pET-Blue2 expression system as previously described [12]. The purity of IscR protein was more than 90% as judged by a major band corresponding to the 18 kDa protein observed in SDS-PAGE. Gel mobility shift assays were performed as previously described [44] using a ^32^P-labeled probe containing either wild type or mutagenized *nfuA* promoter in which putative IscR binding site was mutated. The probe was amplified from pP_*nfuA*_ (for wild-type *nfuA* promoter) or pP*_nfuA_ (for mutagenized *nfuA* promoter) with ^32^P-labeled BT2879 and BT3291 primers. Binding reactions consisting of 3 fmol of labeled probe in 25 μl of reaction buffer containing 20 mM Tris-HCl (pH 8.0), 50 mM KCl, 4 mM MgCl_2_, 0.5 mM EDTA, 0.02 mg ml^-1^ bovine serum albumin (BSA), 5 mM dithiothreitol (DTT), 10% (v/v) glycerol, 200 ng of poly(dI-dC) and varied concentrations of purified IscR were incubated at 25°C for 20 min. Protein-DNA complexes were separated by electrophoresis on a 7% nondenaturing PAGE in 0.5x Tris-borate-EDTA buffer at 4°C and visualized by exposure to X-ray film.

### qRT-PCR

Reverse transcription and real-time PCR (qRT-PCR) was performed as previously described [6, 45]. Essentially, total RNA was extracted from uninduced and stress induced cultures. After DNase treatment, the RNA was reversed transcribed before 10 ng cDNA was added into a KAPA SYBR^®^ FAST qPCR kit containing specific primer pair (BT2841 and BT2860 for *nfuA*, BT3186 and BT3187 for *iscR*, BT3555 and BT3556 for *fdx2*, BT3046 and BT3047 for *soxR*, BT3351 and BT3352 for *PA2274* and BT2781 and BT2782 for *16S rRNA* as an internal control). The reactions run on Applied Biosystems StepOnePlus^TM^ under the following conditions: denaturation at 95°C for 10 s, annealing at 60°C for 30 s, and extension at 60°C for 30 s, for 40 cycles. Relative expression was calculated using STEPONE software v2.1 and expressed as folds of expression relative to the level of PAO1 wild type grown under uninduced condition. The experiments were independently repeated three times and the means ± standard deviations (SD) are shown.

### Plate sensitivity assay

The oxidant resistance level was determined using a plate sensitivity assay as previously described [45]. Briefly, 10 μl of each dilution of 10-fold serially diluted exponential phase cells (adjusted to give OD_600_ of 0.1) was spotted onto LB agar plate containing appropriate concentrations of testing agents. The surviving colonies were scored after overnight incubation at 37°C. The resistance level for each agent was expressed as the % survival, defined as the percentage of the colony forming units (CFU) on plates containing testing agent over the CFU on plates without testing agent.

### Enzymatic assays

Crude cell lysate preparation and total protein determination were performed as previously described [46]. Succinate dehydrogenase activity assay was carried out as previously described and enzyme activity was expressed as ΔA_600_ per min per milligram protein [47]. Aconitase activity was determined using Abcam’s Aconitase Assay Kit (ab83459). One unit of aconitase is defined as the amount of enzyme that isomerizes 1.0 μmol of citrate to isocitrate per min at pH 7.4 at 25°C. Nitrate reductase activity was monitored by measuring the reduction of nitrate to nitrite with methyl viologen as the electron donor as previously described [48]. One unit of activity is defined as the amount of enzyme capable of producing 1 nmol of NO_2_ per min at 25°C.

### Nematode killing assays

All experiments using *Caenorhabditis elegans* were conducted following the protocol approved by the Institutional Animal Care and Use Committee of the Chulabhorn Research Institute (CRI-IACUC).

The virulence of *P*. *aeruginosa* strains was evaluated using a *Caenorhabditis elegans* model host system; both slow and fast killing experiments were conducted as previously described [12, 35, 49]. The fourth larval (L4) stage worms (approximately 30 animals per plate) were used to test the virulence of *P*. *aeruginosa* strains. Worm killing was scored as dead if touching-reflected movement was not detected under dissecting microscope. Scoring was done after 16, 24, 40, 48 and 64 h for fast killing and 1, 2, 3, 4, and 5 days for slow killing experiments. The experiments were carried out in blind fashion and data shown were analyzed from nine independently biological replicates.

### Statistics

Group data are presented as means ± standard deviation (SD). The Student’s *t*-test was used to determine differences between means using the function of Excel (Microsoft, Washington) and the SPSS (version 17.0; SPSS Inc.) statistical package. Unless otherwise is stated, *p* values of < 0.05 were considered significant.

## Supporting information

S1 Fig Expression and Western analyses of *nfuA* in *P*. *aeruginosa* strains. (A) Western analysis of mutated NfuA proteins. Crude protein extracts from exponential-phase cultures of the Δ*nfuA* mutant carrying miniTn7T containing 6His-tag NfuA-WT, C152S, C155S, C43,47S or a vector control (Tn7T) were partially purified using Ni-NTA column prior to loading (40 mg) onto SDS-PAGE. Western blot was performed using anti-6His antibody conjugated with HRP. (B) *nfuA* expression analysis. End-point reverse transcription PCR was performed using BT2841 and BT2842 primers and cDNAs was prepared from total RNA samples extracted from *P*. *aeruginosa* strain as templates.
(TIF)
