# Supplementary figures and images for: Pseudomonas aeruginosa nfuA: Gene regulation and its physiological roles in sustaining growth under stress and anaerobic conditions and maintaining bacterial virulence
Source: PLoS One. 2018 Aug 9;13(8):e0202151. doi: 10.1371/journal.pone.0202151 (PMC6084964; doi:10.1371/journal.pone.0202151)

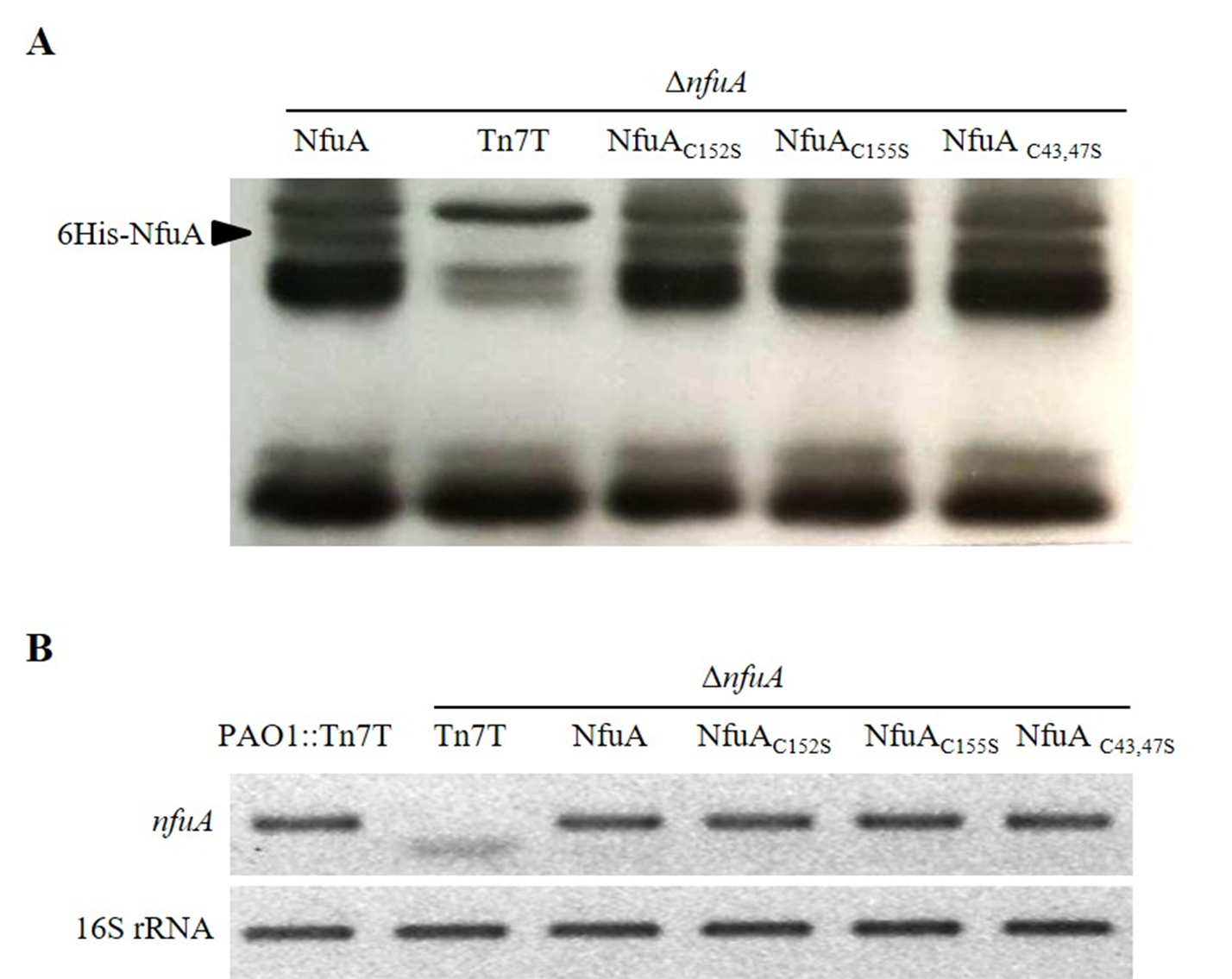

Supplement: S1 Fig — (A) Western analysis of mutated NfuA proteins. Crude protein extracts from exponential-phase cultures of the ΔnfuA mutant carrying miniTn7T containing 6His-tag NfuA-WT, C152S, C155S, C43,47S or a vector control (Tn7T) were partially purified using Ni-NTA column prior to loading (40 mg) onto SDS-PAGE. Western blot was performed using anti-6His antibody conjugated with HRP. (B) nfuA expression analysis. End-point reverse transcription PCR was performed using BT2841 and BT2842 primers and cDNAs was prepared from total RNA samples extracted from P. aeruginosa strain as templates. (TIF) [file pone.0202151.s001.tif]
